# Esophageal schwannoma: a case report

**DOI:** 10.1186/1477-7819-11-253

**Published:** 2013-10-02

**Authors:** Masahiro Kitada, Yoshinari Matsuda, Satoshi Hayashi, Kei Ishibashi, Kensuke Oikawa, Naoyuki Miyokawa

**Affiliations:** 1 Department of Surgery, Asahikawa Medical University, Midorigaoka-Higashi 2-1-1-1, Asahikawa, Hokkaido 078-8510, Japan; 2 Department of Clinical Pathology, Asahikawa Medical University, Asahikawa, Japan

## Abstract

Most tumorous lesions of the esophagus are esophageal cancers. Benign primary tumors of the esophagus are uncommon, and account for approximately 2% of all esophageal tumors. More than 80% of benign esophageal tumors are leiomyomas, with schwannomas being rare. A 55-year-old woman visited our internal medicine department with complaints of palpitations and discomfort during swallowing. A chest computed tomography scan showed a lobulated tumor (75 × 57 × 80 mm) in the upper to middle mediastinum, with homogenous inner opacity, compressing the esophagus. Upper gastrointestinal endoscopy revealed a smooth-surfaced elevated lesion covered with normal mucosa, and a schwannoma was diagnosed based on the biopsy result. The tumor was large. It was thus considered to be difficult to repair the esophagus by direct anastomosis after tumor resection. Therefore, subtotal esophagectomy and esophagogastrostomy in the right thorax were performed. Histopathological examination revealed spindle-shaped cells in a fasciculated and disarrayed architecture and nuclei in a palisading pattern. Immunohistochemical studies revealed S100 protein positivity and the absence of staining for α smooth muscle actin (αSMA), CD34 and CD117, thereby establishing the diagnosis of benign schwannoma. Her postoperative course was uneventful and there has been no evidence of recurrence to date.

## Background

The incidence of benign primary tumors of the esophagus is low. Most are leiomyomas, and schwannomas are rare. We report a patient with schwannoma who was referred to us for evaluation of a mediastinal tumor. Detailed examination yielded a diagnosis of schwannoma arising from the esophageal submucosa and radical surgery was performed.

## Case presentation

**Figure 1:**
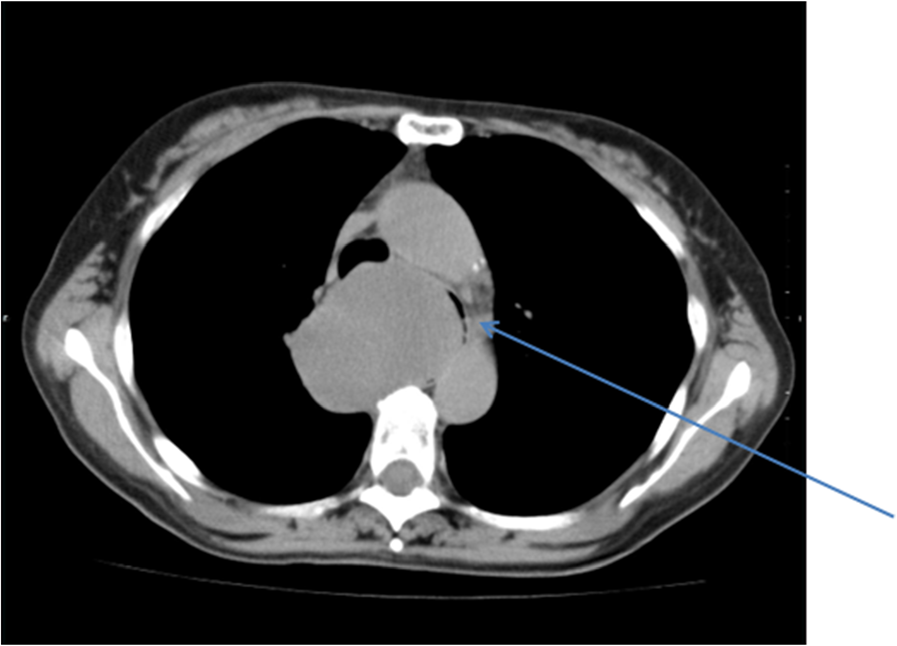
**Chest computed tomography (CT) scan showed a lobulated tumor (75 × 57 × 80 mm) in the upper to middle mediastinum.** The arrow shows compression of the esophagus.

**Figure 2:**
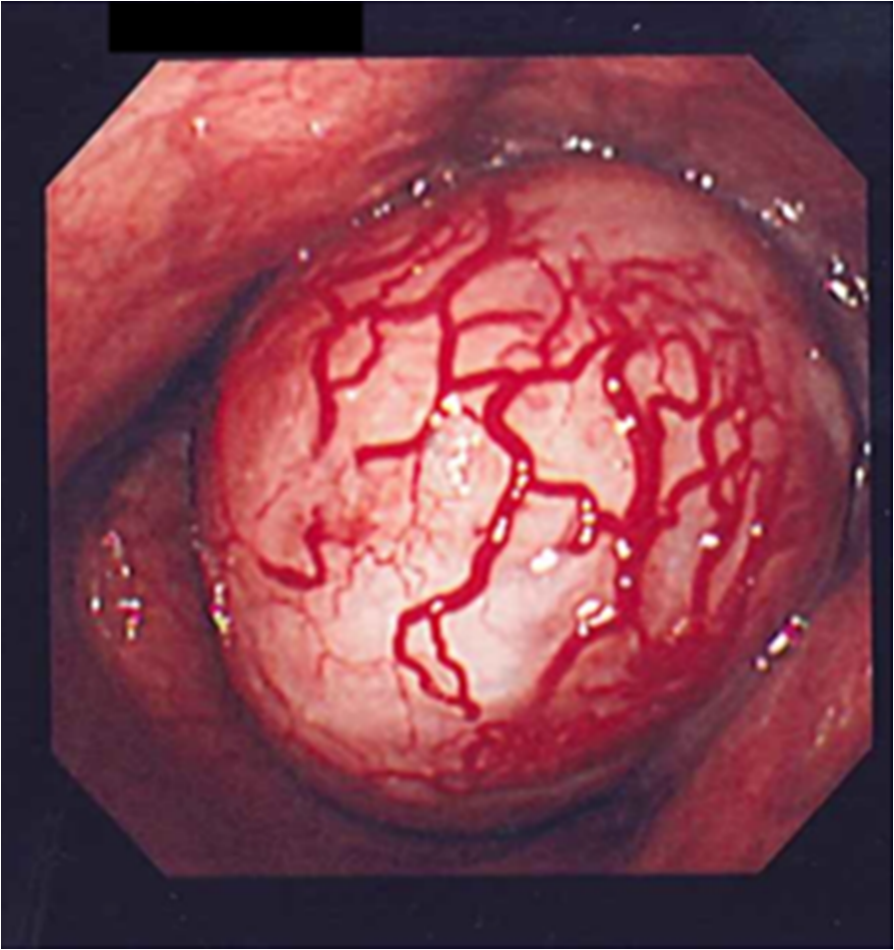
**Upper gastrointestinal endoscopy showing a smooth elevated lesion, 22 cm from the incisor teeth.** A mucous membrane was accompanied by the venous dilation.

**Figure 3:**
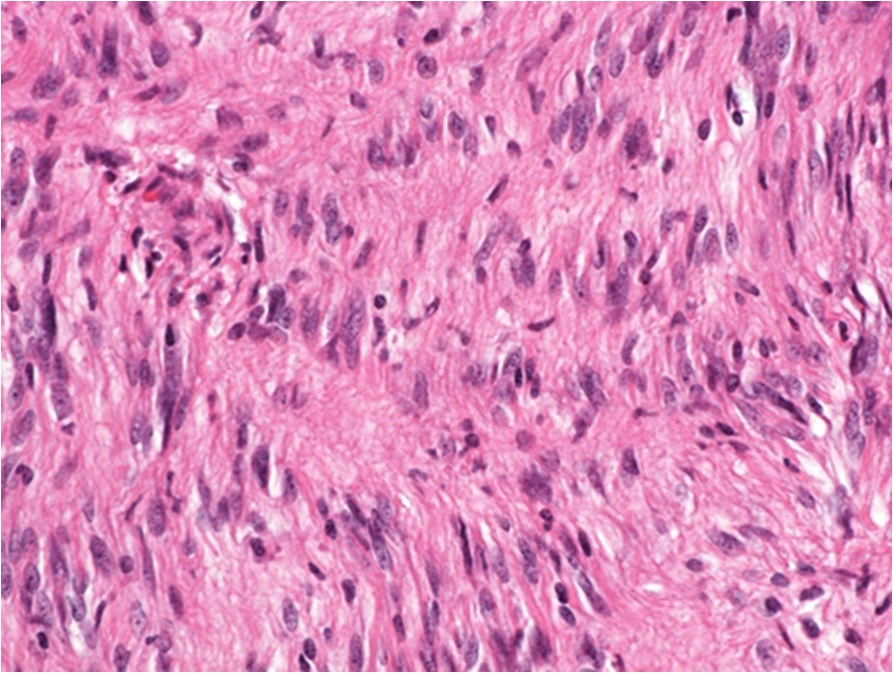
Histopathological findings revealed spindle-shaped cells in a fasciculated and disarrayed architecture and nuclei in a palisading pattern (hematoxylin and eosin stain, ×400).

**Figure 4:**
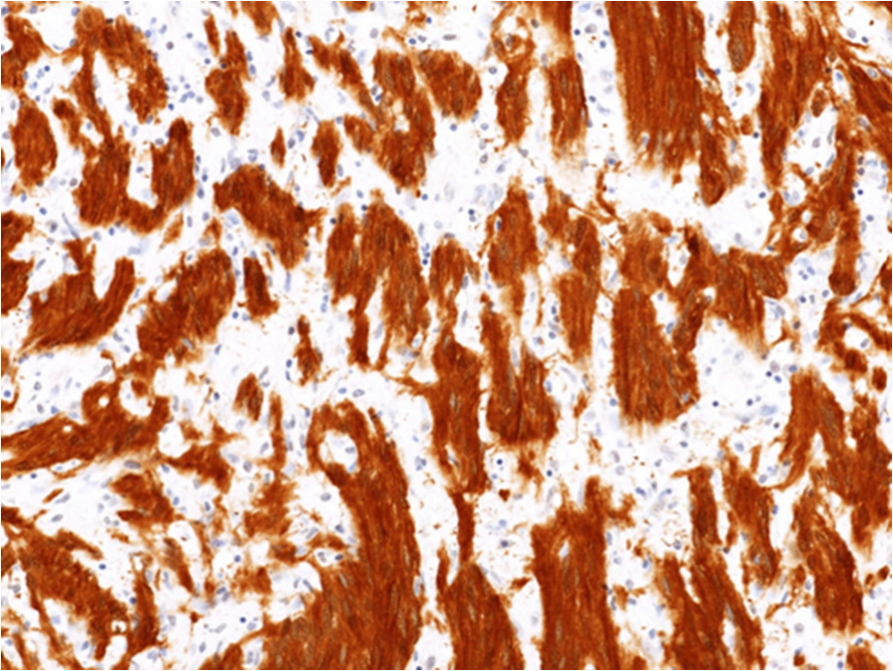
Immunohistochemical studies revealed S100 protein positivity (×200).

A 55-year-old woman visited our hospital with complaints of palpitations and discomfort during swallowing. Her medical and familial histories were unremarkable. A frontal chest radiograph showed a smooth round mass, and a lateral radiograph showed a smooth mass slightly larger than 7 cm in diameter in the middle mediastinum between the trachea and the vertebral bodies. A chest computed tomography (CT) scan showed a lobulated tumor (75 × 57 × 80 mm) in the upper to middle mediastinum, with a homogenous inner component, compressing the esophagus (Figure 1). Magnetic resonance imaging (MRI) of the chest revealed no invasion of surrounding organs. On imaging studies, a mesenchymal tumor such as gastrointestinal stromal tumor was suspected. Upper gastrointestinal endoscopy showed a smooth elevated lesion, 22 cm from the incisor teeth. A mucous membrane was accompanied by the venous dilation (Figure 2). A schwannoma was diagnosed based on the biopsy result for the lesion. Although there was no evidence of malignancy, the patient underwent surgery because of the large size of the tumor, dysphagia, palpitations caused by the tumor compressing the heart, and a suspicion of malignant potential. The patient was placed in the left lateral position and underwent a mini thoracotomy via the fifth right intercostal space with thoracoscopic assistance. A mass slightly larger than 8 cm in diameter was found adjacent to the mid-thoracic esophagus. From above, the tissue surrounding the esophagus was detached and the tumor was excised. The resulting defect in the adventitia and muscular layer was extensive. A direct anastomosis was considered to be difficult, such that subtotal esophagectomy and esophagogastrostomy in the right thorax were performed. The resected specimen showed normal esophageal mucosa. The tumor was well demarcated and elastic hard, had a globular appearance, and measured 75 × 57 × 80 mm. The cut surface was almost uniformly milky white. Histopathological examination revealed spindle-shaped cells in a fasciculated and disarrayed architecture and nuclei in a palisading pattern (Figure 3). Immunohistochemical studies revealed S100 protein positivity (Figure 4) and the absence of staining for α smooth muscle actin (αSMA), CD34 and CD117, establishing the diagnosis of benign schwannoma. Her postoperative course was uneventful and there has been no evidence of recurrence to date.

## Discussion

Most tumorous lesions of the esophagus are esophageal cancers. Benign primary tumors of the esophagus are uncommon, and account for approximately 2% of all esophageal tumors. More than 80% of benign esophageal tumors are leiomyomas, with Schwannomas being rare [1,2]. Schwannoma of the esophagus more frequently develops in women than in men and these tumors are often located in the upper and mid esophagus in the mid mediastinum. A preoperative diagnosis of this condition is difficult, and the definitive diagnosis is often established after resection [3]. A schwannoma that developed in the posterior mediastinum was reported [4]. Furthermore, a patient with a malignant schwannoma has also been reported, but such cases are extremely rare [5].

Symptoms of this disease include dysphagia, dyspnea, and chest pain, and are likely to appear and worsen as the schwannoma increases in size [6]. Fluorodeoxyglucose positron emission tomography as well as CT and MRI are reportedly useful for the confirmation of mediastinal tumors [7]. However, diagnosing a schwannoma is difficult with only imaging studies. In our present case as well, the results from the biopsy obtained with esophagogastroendoscopy were necessary for establishing the diagnosis. Schwannoma is a submucosal tumor, and endoscopic ultrasonography-guided fine needle aspiration biopsy is reportedly useful for both diagnosis and management [8].

In general, histological features of schwannoma include spindle-shaped tumor cells arranged in a palisading pattern or with loose cellularity in a reticular array. Immunohistochemical stainings for S100 protein, αSMA, CD34 and CD117 are also useful [9].

Chemotherapy and radiation therapy are ineffective such that surgical excision including the capsule is indicated for symptomatic cases and also for those with possibly malignant schwannoma. The use of enucleation with video-assisted thoracoscopic surgery is becoming common for small tumors (≤2 cm) [3]. However, for large tumors (≥8 cm) with broad areas adjacent to the esophageal muscular layer, the mucosal defect becomes extensive, and esophagectomy and esophagogastrostomy are thus usually performed [10]. In our present case, the tumor was approximately 8 cm in diameter, and the defect of the adventitia and muscular layer was extensive after tumor excision. Thus, direct anastomosis was considered to carry an increased risk for complications including anastomotic leakage and esophageal stricture. Therefore, esophagectomy and esophagogastrostomy were performed. Her postoperative course was uneventful. She had neither postoperative stricture nor problems with eating and drinking. This favorable outcome confirmed the procedure to have been appropriate.

## Conclusions

Here, we report a relatively rare case of schwannoma of the esophagus, initially identified as a large mediastinal tumor. The tumor was 80 mm in diameter and the post-resection defect of the adventitia and muscular layer was extensive. Esophagectomy and esophagogastrostomy were thus performed and good postoperative recovery was achieved.

## Consent

Written informed consent was obtained from the patients for publication of this case report and any accompanying images. A copy of the written consent is available for review by the Editor-in-Chief of this journal.

## Competing interests

The authors declare that they have no competing interests.

## Authors’ contributions

MK operated on this case and analyzed all data. YM, SH and KS assisted with the operation. KO and NM diagnosed the pathology of this case. All authors read and approved the final manuscript.
